# Supplementary material for: Health Services Use and Health Outcomes among Informal Economy Workers Compared with Formal Economy Workers: A Systematic Review and Meta-Analysis
Source: Int J Environ Res Public Health. 2021 Mar 19;18(6):3189. doi: 10.3390/ijerph18063189 (PMC8003536; doi:10.3390/ijerph18063189)
Supplement: Supplementary file 1 [file ijerph-18-03189-s001.pdf]

Table S1. PubMed search terms.

Search	Query
#9	Search (((#5 OR #7))) AND ("1999/01/01"[Date - Publication] : "2020/03/31"[Date - Publication])
#8	Search (#5 OR #7)
#7	Search (#3 AND #6)
#6	Search (occupational injuries[mh] OR occupational injur*[tiab] OR (occupational[tiab] AND (accident*[tiab] OR disease*[tiab] OR disorder*[tiab] OR illness[tiab] OR dysfunction[tiab])) OR work-related injur*[tiab] OR workplace injur*[tiab] OR work place injur*[tiab] OR back pain*[tiab] OR limb pain*[tiab] OR neck pain*[tiab] OR leg pain*[tiab] OR fatal injur*[tiab] OR musculoskeletal diseases [mh] OR (musculoskeletal[tiab] AND (complaint*[tiab] OR disease*[tiab] OR disorder*[tiab])) OR orthopedic disorder*[tiab] OR respiratory tract infections[mh] OR respiratory tract infection*[tiab] OR bronchitis[tiab] OR asthma[tiab] OR lung diseases[mh] OR lung disease*[tiab] OR lung disorder*[tiab] OR pneumoconiosis [tiab] OR pneumonitis[tiab] OR pneumonia[tiab] OR tuberculosis[tiab] OR TB[tiab] OR HIV[mh] OR (human immun*[tiab] AND (deficiency virus*[tiab])) OR human immunodeficiency virus[tiab] OR mental disorders[mh] OR mental disorder*[tiab] OR mental disease*[tiab] OR behavior disorder*[tiab] OR behaviour disorder*[tiab] OR psychiatric disorder*[tiab] OR anxiety[mh] OR anxiety[tiab] OR depression[mh] OR depression*[tiab] OR (depressive[tiab] AND (symptom*[tiab] OR disorder*[tiab] OR illness[tiab] OR syndrome[tiab])) OR sleep disorder*[tiab] OR insomnia*[tiab] OR sleeplessness[tiab] OR skin diseases[mh] OR skin disorder*[tiab] OR skin condition*[tiab] OR skin disease*[tiab] OR rash[tiab] OR eczema[tiab] OR dermatitis[tiab] OR dermatosis[tiab] OR (dermal[tiab] AND (disease*[tiab] OR disorder*[tiab])) OR hearing loss, noise induced[mh] OR noise-induced hearing loss[tiab] OR acoustic trauma[tiab] OR acoustic accident[tiab] OR hearing loss[tiab] OR sound injury[tiab] OR cancer*[tiab] OR neoplasms[mh] OR neoplasm*[tiab] OR tumor[tiab] OR tumors[tiab] OR tumour*[tiab] OR occupational health[tiab] OR health hazards[tiab])
#5	Search (#3 AND #4)
#4	Search (health services[mh] OR health service*[tiab] OR (healthcare[tiab] AND (service*[tiab] OR agenc*[tiab])) OR (health services[tiab] AND (service*[tiab] OR agenc*[tiab])) OR health services accessibility[mh] OR access to health services[tiab] OR health services needs and demand[mh] OR traditional heal*[tiab] OR (health[tiab] AND (access*[tiab] OR inaccessib*[tiab] OR use[tiab] OR user[tiab] OR users[tiab] OR utilis*[tiab] OR utiliz*[tiab])) OR (health services[tiab] AND (access*[tiab] OR inaccessib*[tiab] OR use[tiab] OR user[tiab] OR users[tiab] OR utilis*[tiab] OR utiliz*[tiab])) OR (healthcare[tiab] AND (access*[tiab] OR inaccessib*[tiab] OR use[tiab] OR user[tiab] OR users[tiab] OR utilis*[tiab] OR utiliz*[tiab])) OR delivery of health services[mh] OR health services delivery[tiab] OR healthcare system*[tiab] OR health services system*[tiab])
#3	Search (informal sector[mh] OR informal sector*[tiab] OR informal econom*[tiab] OR street vendor*[tiab] OR street hawker*[tiab] OR road side hawker*[tiab] OR road side vendor*[tiab] OR road side shop keeper*[tiab] OR roadside hawker*[tiab] OR roadside vendor*[tiab] OR roadside shop keeper*[tiab] OR waste recycling[tiab] OR waste recycler*[tiab] OR recycling worker*[tiab] OR garbage picker*[tiab] OR waste picker*[tiab] OR informal miner[tiab] OR informal mining[tiab] OR informal miners[tiab] OR artisan* miner[tiab] OR artisan* miners[tiab] OR artisan* mining[tiab] OR informal recycler*[tiab] OR informal recycling[tiab] OR seasonal picker*[tiab] OR seasonal work*[tiab] OR informal enterprise*[tiab] OR informal service sector*[tiab] OR informal work*[tiab] OR informal vendor*[tiab] OR informal employ*[tiab] OR ragpicker*[tiab] OR rag picker*[tiab] OR informal subsistence worker*[tiab] OR scrap recycl*[tiab] OR scrap metal recycl*[tiab] OR scrap worker*[tiab] OR informal trader*[tiab] OR seasonal farm worker*[tiab] OR seasonal farmworker*[tiab] OR seasonal labour*[tiab] OR seasonal labor*[tiab] OR informal waste management[tiab])

Table S2. Risk of Bias Table per Study.

1. Lopez-Bonilla. 2005: Fatal occupational injuries.

**Domain 1: Bias due to selection of participants into the study**	
**Reporting item**	**Assessment**
Definition of target population	*All workers (aged >10 years) in Nicaragua in 2005*
Definition of the study population	*Participants of national household survey collected by national statistics office in Nicaragua in 2005*
Rating	*Probably low*
Justification	*Study sample was probably representative of target population and total population. Assumed that sampling was random, because there was mention of stratified sampling. We had some concerns for risk of bias in the* rate of participation in the outcome assessment due to indirect evidence for underreporting.
**Domain 2: Bias due to lack of blinding of study personnel**	
**Reporting item**	**Your assessment**
Rating	*Probably low*
Justification for rating	*No information on blinding. However, we judge the risk of bias to be probably low, because it is unlikely that study personnel would have biased the results due their knowledge of exposure status, especially because exposure assessment and outcome assessment were done by separate study personnel without knowledge of the objective of the current study. However, data analysts may have still biased the study, but that is unlikely.*
**Domain 3: Bias due to misclassification of the exposure**	
**Reporting item**	**Your assessment**
Rating	*Low*
Justification for rating	*Definition in study meets more than two of our criteria for eligibility for informal economy workers, so definition as per our protocol. Although the definition of domestic worker was not met, Table 3 on P 241 indicated that this population is relatively small.*
**Domain 4: Bias due to misclassification of the outcome**	
**Reporting item**	**Your assessment**
Rating	*Probably high*
Justification for rating	*No agreed reporting definitions. High risk of underreporting of fatal injuries. Direct evidence for misreporting of outcome. Table 1 shows variable reporting of fatal occupational injuries across sources (P239)*
**Domain 5: Bias due to incomplete exposure data**	
**Reporting item**	**Your assessment**
Rating	*No information*
Justification for rating	No information provided in study record.
**Domain 6: Bias due to incomplete outcome data**	
**Reporting item**	**Your assessment**
Rating	*Probably high*
Justification for rating	*Unknown, but likely large proportion of missing outcome data in at least one sector (agriculture) that is the largest sector (see Table 3), Page 240.*
**Domain 7: Bias due to selective reporting of exposures** Could relevant exposures or exposure (informal economy work) categories be selectively not reported?	
**Reporting item**	**Your assessment**
Rating	*Probably low*
Justification for rating	*Exposure were the informal sector- used censored data.* Workers defined as working in the informal economy were defined on Page 239.

Domain 8: Bias due to selective reporting of outcome Could relevant outcome or outcome categories be selectively not reported?	
**Reporting item**	**Your assessment**
Rating	*High*
Justification for rating	Under reporting in some sectors – P240
**Domain 9: Bias due to differences between numerator and denominator**	
**Reporting item**	**Your assessment**
Rating	*Probably low*
Justification for rating	*Census used as a denominator (numerator should be part of the denominator)*
**Domain 10: Conflict of interest**	
**Reporting item**	**Your assessment**
Rating	*Probably low*
Justification for rating	*Declaration by authors, no other evidence of conflict Page 238*

2. Mora, 2011: Fatal occupational injuries.

**Domain 1: Bias due to selection of participants into the study**	
**Reporting item**	**Your assessment**
Definition of target population	*All formal and informal workers*
Definition of the study population	*Formal and informal workers with occupational injuries*
Rating	*Probably Low*
Justification	*This was secondary data analyses using multiple sources of data. There is a possible risk of missing. Selection bias is low because any death due to occupational injury will be included if the incident was reported. See page 244.*
**Domain 2: Bias due to lack of blinding of study personnel**	
**Reporting item**	**Your assessment**
Rating	*Probably Low*
Justification for rating	*No information on blinding. However, we judge the risk of bias to be probably low, because it is unlikely that study personnel would have biased the results due their knowledge of exposure status, especially because exposure assessment and outcome assessment were done by separate study personnel without knowledge of the objective of the current study.*
**Domain 3: Bias due to misclassification of the exposure**	
**Reporting item**	**Your assessment**
Rating	*Probably Low*
Justification for rating	*Definition in study meets more than two of our criteria for eligibility for informal economy workers, however informality was inferred if people did not have insurance against risks at work, so it is possible that some were classified as informal even if they were not. See page 244 and 245*
**Domain 4: Bias due to misclassification of the outcome**	
**Reporting item**	**Your assessment**
Rating	*Probably low*
Justification for rating	*Appropriately defined outcome. Page 244.*
**Domain 5: Bias due to incomplete exposure data**	
**Reporting item**	**Your assessment**
Rating	*No information*
Justification for rating	*No information provided in study record.*

Domain 6: Bias due to incomplete outcome data	
**Reporting item**	**Your assessment**
Rating	*High*
Justification for rating	*Missing outcome data in agricultural sector, home based workers and the self-employed is likely. On page 247- the authors state: "the major problem in the FOI data in Costa Rica is the lack of identification and registration system of occupational injuries among the informal economy workers, many of whom work at home, which is not necessarily considered a worksite."*
**Domain 7: Bias due to selective reporting of exposures**	
**Reporting item**	**Your assessment**
Rating	*Probably low*
Justification for rating	*Exposures were the informal sector data obtained from surveillance systems so more likely missing data and not selective reporting. See page 245.*
**Domain 8: Bias due to selective reporting of outcome**	
**Reporting item**	**Your assessment**
Rating	*Probably High*
Justification for rating	*Under reporting in some sectors*
**Domain 9: Bias due to differences between numerator and denominator**	
**Reporting item**	**Your assessment**
Rating	*Probably low*
Justification for rating	*National secondary data used as a numerator and census data used as denominator*
**Domain 10: Conflict of interest**	
**Reporting item**	**Your assessment**
Rating	Probably low
Justification for rating	*Disclosure statement made. No evidence of conflict of interest*

3. Calys-Tagoe, 2017: Non-fatal occupational injuries.

**Domain 1: Bias due to selection of participants into the study**	
**Reporting item**	**Your assessment**
Definition of target population	*All artisanal and small scale miners*
Definition of the study population	*Artisanal and small scale miners- licensed and unlicensed*
Rating	*Probably high*
Justification	*Unlicensed mine workers may not have been cooperative and thus not participated in the study. Also they were not randomly selected from a sampling frame*
**Domain 2: Bias due to lack of blinding of study personnel**	
**Reporting item**	**Your assessment**
Rating	*Probably low*
Justification for rating	*No information provided but since trained fieldworkers were used- bias should be limited.*<br>*The interviews were conducted by indigenes of the study area who were well versed in both English and Twi (the local dialect), had completed a minimum of secondary education and were trained specifically for this study. Each completed questionnaire was cross-checked by the Principal Investigator on the field to ensure completeness (from 2015 research paper in IJERPH).*
**Domain 3: Bias due to misclassification of the exposure**	
**Reporting item**	**Your assessment**

Rating	*No information*
Justification for rating	*The two groups were recruited differently and from two different types of mining sectors (unlicensed and licensed mines). The study compared licensed artisanal mines, not miners so it is possible that some of the miners on the licensed mines could have been in informal employment.*
**Domain 4: Bias due to misclassification of the outcome**	
**Reporting item**	**Your assessment**
Rating	*Probably high*
Justification for rating	*Self-reported injuries. Injuries not defined in paper*
**Domain 5: Bias due to incomplete exposure data**	
**Reporting item**	**Your assessment**
Rating	*Low*
Justification for rating	*Exposure was unlicensed mine (informal economy worker) and licensed (formal economy worker) and these workers were recruited at specific sites thus unlikely to be incomplete. Page 2.*
**Domain 6: Bias due to incomplete outcome data**	
**Reporting item**	**Your assessment**
Rating	*Probably high*
Justification for rating	*Recall bias is possible since self-reported, under reporting is also a possibility*
**Domain 7: Bias due to selective reporting of exposures**	
**Reporting item**	**Your assessment**
Rating	*Low*
Justification for rating	*Recruited from specific sites so unlikely.*
**Domain 8: Bias due to selective reporting of outcome**	
**Reporting item**	**Your assessment**
Rating	*Probably low*
Justification for rating	*Under reporting is possible but maybe similar for the licensed and unlicensed workers*
**Domain 9: Bias due to differences between numerator and denominator**	
**Reporting item**	**Your assessment**
Rating	*Probably high*
Justification for rating	*Denominators for the unlicensed mines were not known- so rates were calculated for person years. See page 7. "While it has been estimated that upwards of 85% of Ghana's ASGM workers do not have licenses [19], we were unable to properly enumerate this in the study region though we have no reason to believe that the selected mines were atypical."*
**Domain 10: Conflict of Interest**	
**Reporting item**	**Your assessment**
Rating	*Probably low*
Justification for rating	*No evidence*

4. Cunningham, 2012: Non-fatal occupational injuries.

**Domain 1: Bias due to selection of participants into the study**	
**Reporting item**	**Your assessment**
Definition of target population	*People working in the waste picking , recycling industry*
Definition of the study population	*Informal = Ganchero's who work in landfills ; formal= recyclers working for an NGO*
Rating	*Probably high*
Justification	*Refusal rate maybe higher and no data to compare those that refused compared with those that participated. Sampling was not random.*<br>*The formal worker sample was very low (n=12)*<br>*Page 182: "Our sample represents approximately one-fifth of the ganchero workforce (estimated at 500 workers …). Our refusal rate was 5% (five refusals); however this might be an underestimation because of poor documentation …"*
**Domain 2: Bias due to lack of blinding of study personnel**	
**Reporting item**	**Your assessment**
Rating	*Probably low*
Justification for rating	*No information provided but since trained research staff were used- bias should be limited.*
**Domain 3: Bias due to misclassification of the exposure**	
**Reporting item**	**Your assessment**
Rating	*low*
Justification for rating	*The two groups were recruited from two specific sites thus there is little risk of misclassification.*<br>*"A cross-section of 102 gancheros were surveyed on four separate weekdays in August 2009 at the Cateura landfill in Asunció n. Participants included any informal workers at the landfill on the four survey days. Our sample represents approximately one-fifth of the ganchero workforce (estimated at 500 workers by the leader of the gancheros co-operative). Our refusal rate was 5% (five refusals); however, this may be an underestimation because of poor documentation on the first survey day when one of the surveyors did not document refusals. For our comparison group, workers at the NGO Procycla, we consented 12 out of 12 workers with no refusals."*
**Domain 4: Bias due to misclassification of the outcome**	
**Reporting item**	**Your assessment**
Rating	*Probably low*
Justification for rating	*Outcome data was determined by a questionnaire administered by trained interviewers in a language of the subjects and the outcomes were straight forward – thus outcomes unlikely to be misclassified*
**Domain 5: Bias due to incomplete exposure data**	
**Reporting item**	**Your assessment**
Rating	low
Justification for rating	*Exposure was the informal and formality of worker. The workers were recruited by the PI. Thus unlikely risk of incomplete exposure data.*<br>*Page 182: "Participants included any informal workers at the landfill on the four survey days. For our comparison group, workers at the NGO Procycla, we consented 12 out of 12 workers with no refusals. The principal investigator and two trained surveyors obtained informed consent from participants using an oral script."*
**Domain 6: Bias due to incomplete outcome data**	

Reporting item	Your assessment
Rating	*Probably high*
Justification for rating	*Recall bias is possible since self-reported, under reporting is also a possibility*
**Domain 7: Bias due to selective reporting of exposures**	
**Reporting item**	**Your assessment**
Rating	*Low*
Justification for rating	*Recruited from specific sites so unlikely.*<br><br>*"Page 182: Participants included any informal workers at the landfill on the four survey days. For our comparison group, workers at the NGO Procycla, we consented 12 out of 12 workers with no refusals. The principal investigator and two trained surveyors obtained informed consent from participants using an oral script."*
**Domain 8: Bias due to selective reporting of outcome**	
**Reporting item**	**Your assessment**
Rating	*Probably high*
Justification for rating	*Under reporting is possible and only 12 formal employees, so not very representative.*
**Domain 9: Bias due to differences between numerator and denominator**	
**Reporting item**	**Your assessment**
Rating	*Probably high*
Justification for rating	*Denominators very low- See page 182*
**Domain 10: Conflict of interest**	
**Reporting item**	**Your assessment**
Rating	*Probably low*
Justification for rating	*No evidence. Page 187. Declaration of no conflict*

5. Santana, 2003: Non-fatal occupational injuries.

**Domain 1: Bias due to selection of participants into the study**	
**Reporting item**	**Your assessment**
Definition of target population	*All workers in Brazil*
Definition of the study population	*Inhabitants from City of Salvador 18-65 years old who reported having a paid job*
Rating	*Probably low*
Justification	*Random sampling, this was a community-based study with a random cluster area sample of the inhabitants from the city of Salvador, capital of the State of Bahia. (page 1 of translated doc)*
**Domain 2: Bias due to lack of blinding of study personnel**	
**Reporting item**	**Your assessment**
Rating	*Probably low*
Justification for rating	*Trained fieldworkers used- supervised by teachers involved in research*
**Domain 3: Bias due to misclassification of the exposure**	
**Reporting item**	**Your assessment**
Rating	*Probably low*
Justification for rating	*Not defined in detail but acceptable.*<br><br>*"identified the people who had gainful employment or work household, for the family." Page 8*<br>*"All occupations in the period were recorded, the main one being identified as by the interviewee himself, who was that used the highest remuneration criteria or greater proportion of allotted time." Page 10*<br>*"The existence or not of formal contract of employment was analyzed observing the registration in work permit. Due to the peculiarities of the contractual relationship, officials public, entrepreneurs, and professionals." Page 10*
**Domain 4: Bias due to misclassification of the outcome**	
**Reporting item**	**Your assessment**
Rating	*low*
Justification for rating	*Up to the interviewer to decide if occupational or not. Interviewers were experienced.*<br><br>*"As was anticipated that the perception and recognition of accidents occupational hazards by workers in the informal economy was hampered by the of a social security vision, demarcated for administrative and compensatory purposes employed by the national system of social security, aimed at workers formally contracted, it was decided to an indirect approach. In other words, initially "you have suffered an accident, from any nature, ever in life? ";*

	The period referred to in last twelve months. If so, the respondent to specify the circumstances in which it had occurred. When the accident had occurred during the of work activities, or during the trip for work, a questionnaire specific case, in which there was a brief narrative of the event, using their own words of the interviewee. Thus, no direct questions were asked about accident, with the inference being that on the occurrence of an accident at work under the responsibility of the interviewer, which was trained for this task, based on the circumstances described. When there was reference more than one accident in the reference period of the study, it was considered only the most recent". Page 9
**Domain 5: Bias due to incomplete exposure data**	
**Reporting item**	**Your assessment**
Rating	*Probably low*
Justification for rating	*Trained interviewers used*
**Domain 6: Bias due to incomplete outcome data**	
**Reporting item**	**Your assessment**
Rating	*Probably low*
Justification for rating	*Trained interviewers used. "Data collection was performed by interviewers trained, supervised by trainees and teachers involved in the research". Page 7 of translated doc.*
**Domain 7: Bias due to selective reporting of exposures**	
**Reporting item**	**Your assessment**
Rating	*Probably low*
Justification for rating	*Trained interviewers used*
**Domain 8: Bias due to selective reporting of outcome**	
**Reporting item**	**Your assessment**
Rating	*Probably high*
Justification for rating	*Those in the informal economy may not have reported accurately.*<br><br>*"In addition, informal, self-employed or skilled workers, for example, may not be very clear about the notion that are subject to occupational accidents, a concept broadly social security in our society, which would lead to the omission of information. It is therefore possible that these results have been underestimated, since the period of the study (one year) is prolonged, although some care has been taken to reduce these errors" (page 27)*
**Domain 9: Bias due to differences between numerator and denominator**	
**Reporting item**	**Your assessment**
Rating	*low*

Justification for rating	*Numerator is part of the denominator*
**Domain 10: Conflict of interest**	
**Reporting item**	**Your assessment**
Rating	*Probably low*
Justification for rating	*No evidence of conflicts of interest*

6. Da Silva, 2006: Depression.

**Domain 1: Bias due to selection of participants into the study**	
**Reporting item**	**Your assessment**
Definition of target population	*All ragpickers and community members 18 years old and older*
Definition of the study population	*Ragpickers in Pelotas and non ragpickers (community members) living in the same neighbourhood from March to July 2004*
Rating	*Probably high*
Justification	*Method of sampling of ragpickers- convenient, snowballing; also controls that were sampled were those living next to a selected ragpicker = possible selection bias.*<br><br>*"A cross-sectional study was carried out among ragpickers and neighborhood matched referents from March to July 2004.*<br>*Page 2*<br>*Ragpickers were also identified in cooperatives of recycled materials, by key-informants in poor neighborhoods, and by ragpickers who led us to others doing the same work in the neighborhoods where they lived. The referent group was composed of other workers that lived in the same neighborhoods as the ragpickers. To locate referents, interviewers went to neighboring houses,*<br>*starting from those immediately adjacent to each subject's home, in order to locate a suitable non-ragpicker to interview*<br>*Page 2*
**Domain 2: Bias due to lack of blinding of study personnel**	
**Reporting item**	**Your assessment**
Rating	*Probably low*
Justification for rating	*Trained fieldworkers used.*
**Domain 3: Bias due to misclassification of the exposure**	
**Reporting item**	**Your assessment**
Rating	*Probably high*

Justification for rating	*The exposure is informal (ragpickers) vs formal (non ragpickers). However, from the job titles some of the participants considered formal could be from the informal economy such as day labourers and domestic workers*<br><br>*"Domestic work (28.0%), day laborers (33.4%) retail sales (14.6%) and construction (13.4%) were the most frequently reported occupations of the non-ragpickers neighborhood referents" Page 6*
**Domain 4: Bias due to misclassification of the outcome**	
**Reporting item**	**Your assessment**
Rating	*Probably low*
Justification for rating	*Used a validated screening tool for measurement of the outcome. Since it's a screening tool maybe some misclassification but will apply to both groups equally.*<br><br>*"The SRQ-20 has been shown to be a cost-effective way to evaluate mental health in developing countries. It has high validity and well over ten years' history of application in at least 20 countries". Page 3*
**Domain 5: Bias due to incomplete exposure data**	
**Reporting item**	**Your assessment**
Rating	*Probably low*
Justification for rating	*Researchers were able to identify ragpickers and non ragpickers.*<br><br>*"Ragpickers were also identified in cooperatives of recycled materials, by key-informants in poor neighborhoods, and by ragpickers who led us to others doing the same work in the neighborhoods where they lived. The referent group was composed of other workers that lived in the same neighborhoods as the ragpickers. To locate referents, interviewers went to neighboring houses, starting from those immediately adjacent to each subject's home, in order to locate a suitable non-ragpicker to interview." Page 2*
**Domain 6: Bias due to incomplete outcome data**	
**Reporting item**	**Your assessment**
Rating	*Low*
Justification for rating	*All included participants have srq20 results. No missing outcome results mentioned.*
**Domain 7: Bias due to selective reporting of exposures**	
**Reporting item**	**Your assessment**
Rating	*Probably low*
Justification for rating	*Unlikely*
**Domain 8: Bias due to selective reporting of outcome**	

Reporting item	Your assessment
Rating	*Low*
Justification for rating	*Cut offs have been established for this validated tool so unlikely.*<br><br>*"The SRQ-20 has been shown to be a cost-effective way to evaluate mental health in developing countries. It has high validity and well over ten years' history of application in at least 20 countries." Page 3*
**Domain 9: Bias due to differences between numerator and denominator**	
**Reporting item**	**Your assessment**
Rating	*Probably low*
Justification for rating	*Prevalence calculated by using the no. with the outcome over the total sample. So same denominator used for ragpickers and non ragpickers.*
**Domain 10: Conflict of interest**	
**Reporting item**	**Your assessment**
Rating	*Probably low*
Justification for rating	*No evidence. Page 9- declaration*

7. Abbas, 2013: Depression.

**Domain 1: Bias due to selection of participants into the study**	
**Reporting item**	**Your assessment**
Definition of target population	*Cleaners in government hospitals in Zagazig City, Egypt*
Definition of the study population	*Temporary and permanently employed cleaners in 4 government hospitals in the city in Egypt in March 2012*
Rating	*Probably low*
Justification	*Stratified random sampling was used in the temporary and permanent employees.*<br><br>*"A cross-sectional study was conducted on 242 adult temporary cleaners and 209 permanent cleaners working in 4 governmental hospitals in Zagazig City, Sharqia Governorate, Egypt". Page 13*<br><br>*"A stratified random sampling technique was used; where 197 men and 45 women were included in the study. Another group of adult permanent cleaners (n=209) including 171 men and 38 women who were hired for a period of two consecutive years or more were included in the study as the comparison group." Page 15*
**Domain 2: Bias due to lack of blinding of study personnel**	

Reporting item	Your assessment
Rating	*Probably low*
Justification for rating	*List of workers in both groups were known and distributed according to the exposure (temporary and permanent) for random sampling so unlikely to have selection bias.*
**Domain 3: Bias due to misclassification of the exposure**	
**Reporting item**	**Your assessment**
Rating	*Low*
Justification for rating	*List of workers in both groups were known and distributed according to the exposure (temporary and permanent) for random sampling.*<br>*A stratified random sampling technique was used; where 197 men and 45 women were included in the study. Another group of adult permanent cleaners (n=209) including 171 men and 38 women who were hired for a period of two consecutive years or more were included in the study as the comparison group. Page 15*
**Domain 4: Bias due to misclassification of the outcome**	
**Reporting item**	**Your assessment**
Rating	*Low*
Justification for rating	*Known validated instrument used to diagnose CMD*<br>*"Participants were assessed for CMDs using the self-reporting questionnaire 20 items (SRQ-20) that was recommended by the WHO. It has been used by many researchers as a screening instrument for psychiatric epidemiological studies." Page 15*
**Domain 5: Bias due to incomplete exposure data**	
**Reporting item**	**Your assessment**
Rating	Low
Justification for rating	*Results are shown by exposure. Exposure (job status) was known at the time pf participation so unlikely to be incomplete.*<br>*"The total number of adult temporary hired cleaners (18–60 years) in the four studied hospitals at the time of the study was 500 workers. Another group of adult permanent cleaners (n=209) including 171 men and 38 women who were hired for a period of two consecutive years or more were included in the study as the comparison group". Page 15*
**Domain 6: Bias due to incomplete outcome data**	
**Reporting item**	**Your assessment**
Rating	*No information*

Justification for rating	*No information*
**Domain 7: Bias due to selective reporting of exposures**	
**Reporting item**	**Your assessment**
Rating	*Low*
Justification for rating	*Exposures known at time of participation so unlikely.*<br><br>*"The total number of adult temporary hired cleaners (18–60 years) in the four studied hospitals at the time of the study was 500 workers. Another group of adult permanent cleaners (n=209) including 171 men and 38 women who were hired for a period of two consecutive years or more were included in the study as the comparison group". Page 15*
**Domain 8: Bias due to selective reporting of outcome**	
**Reporting item**	**Your assessment**
Rating	*Probably low*
Justification for rating	*Validated tool used. However, it is self-reported and is a screening tool so diagnosis is not definitive. The bias would have affected both groups.*<br><br>*"Participants were assessed for CMDs using the self-reporting questionnaire 20 items (SRQ-20) that was recommended by the WHO. It has been used by many researchers as a screening instrument for psychiatric epidemiological studies." Page 15*
**Domain 9: Bias due to differences between numerator and denominator**	
**Reporting item**	**Your assessment**
Rating	*Low*
Justification for rating	*Denominator included the numerator*<br><br>*See table 1, page 17- numerator and denominators*
**Domain 10: Conflict of interest**	
**Reporting item**	**Your assessment**
Rating	*Probably low*
Justification for rating	*No evidence of conflict of interest*<br><br>*Page 24- declaration*

8. Da Silva, 2006. Musculoskeletal pain.

**Domain 1: Bias due to selection of participants into the study**	
**Reporting item**	**Your assessment**
Definition of target population	*Ragpickers and the general population in the city*
Definition of the study population	*Ragpickers and non ragpickers in the same neighbourhood in Brazil in 2004*
Rating	*Probably high*
Justification	*Method of sampling of ragpickers- convenient, snowballing; also controls that were sampled were those living next to a selected ragpicker = possible selection bias.*<br><br>*"The investigation was based on a large cross-sectional study of ragpickers and neighborhood-matched referents. Data collection took place from March to July 2004, in Pelotas, a city located in the southern-most state of Brazil, Rio Grande do Sul. Pelotas has a population of 338,000." Page 328*
**Domain 2: Bias due to lack of blinding of study personnel**	
**Reporting item**	**Your assessment**
Rating	*Probably low*
Justification for rating	Trained fieldworkers used.
**Domain 3: Bias due to misclassification of the exposure**	
**Reporting item**	**Your assessment**
Rating	*Probably high*
Justification for rating	*The exposure is informal (ragpickers) vs formal (non ragpickers). However, from the job titles some of the participants considered formal could be from the informal economy such as day labourers and domestic workers*<br><br>*"The non-ragpickers reported a number of different kinds of work. The most commonly reported trades were: domestic work (28%), day laborers (33.4%), retail sales (14.6%), and construction (13.4%)." Page 330*
**Domain 4: Bias due to misclassification of the outcome**	
**Reporting item**	**Your assessment**
Rating	*Probably low*
Justification for rating	*There may be a degree of misclassification since its self-reported but this will have occurred in both groups*<br><br>*"Pain symptoms were grouped in three regions according to anatomical proximity of body parts: the lower back, the lower extremities (thighs, lower legs, knees, and ankles) and upper extremities (neck, shoulders, elbows, and wrists/hands)."page 328-329.*

Domain 5: Bias due to incomplete exposure data	
**Reporting item**	**Your assessment**
Rating	*Low*
Justification for rating	*Exposure in each group known at recruitment.*
**Domain 6: Bias due to incomplete outcome data**	
**Reporting item**	**Your assessment**
Rating	*Probably low*
Justification for rating	*Missing data from questionnaire is a possibility but not mentioned and will affect both groups*
**Domain 7: Bias due to selective reporting of exposures**	
**Reporting item**	**Your assessment**
Rating	*Probably low*
Justification for rating	*Unlikely.*<br>*"The investigation was based on a large cross-sectional study of ragpickers and neighborhood-matched referents. Data collection took place from March to July 2004, in Pelotas, a city located in the southern-most state of Brazil, Rio Grande do Sul. Pelotas has a population of 338,000." Page 328*
**Domain 8: Bias due to selective reporting of outcome**	
**Reporting item**	**Your assessment**
Rating	*Probably low*
Justification for rating	*Self-reported questionnaire on Musculoskeletal system pain thus possible low risk.*
**Domain 9: Bias due to differences between numerator and denominator**	
**Reporting item**	**Your assessment**
Rating	*Probably low*
Justification for rating	*Numerator was part of the population used in the denominator.*
**Domain 10: Conflict of interest**	
**Reporting item**	**Your assessment**
Rating	*Probably low*
Justification for rating	*No evidence*

9. Le, 2015: Health services use.

**Domain 1: Bias due to selection of participants into the study**	
**Reporting item**	**Your assessment**
Definition of target population	*Formal and informal migrants and non-migrants in Hanoi, Vietnam*
Definition of the study population	*18-55 year old non migrants and migrants working in industrial zone, PSE and seasonal migrants*
Rating	*Probably high*
Justification	*Non registered migrants may not have been identified correctly by heads of resident groups. Not registered.*<br><br>*"All participants in the study were 18 – 55 years old, which reflects the working age in Viet Nam (18 – 60 for males and 18 – 55 for females). Participants were randomly collected from the sampling frame of each group. The sampling frame of non-migrants was based on the household registration (ho khau) while the sampling frame of migrants came from temporary registration (tam tru) and other non-registered migrants identified by heads of resident groups (to dan pho). "Page 4*
**Domain 2: Bias due to lack of blinding of study personnel**	
**Reporting item**	**Your assessment**
Rating	*Probably low*
Justification for rating	*Trained interviewers used. No information on blinding of study personnel.*<br>*The interviewers in this study were Master's students at the Hanoi School of Public Health and were trained by principal investigators. Page 5*
**Domain 3: Bias due to misclassification of the exposure**	
**Reporting item**	**Your assessment**
Rating	*Probably high*
Justification for rating	*Misclassification of migrant groups may have occurred especially for those considered informal.*<br><br>*"The sampling frame of non-migrants was based on the household registration (ho khau) while the sampling frame of migrants came from temporary registration (tam tru) and other non-registered migrants identified by heads of resident groups (to dan pho)." Page 4*
**Domain 4: Bias due to misclassification of the outcome**	
**Reporting item**	**Your assessment**
Rating	*Probably low*
Justification for rating	*Since self-reported risk of recall bias*

	"Utilization of heath care services was identified as "an ill person who goes to health care centers to seek any treatment (i.e. both in private and public health care centers)". Page 5
**Domain 5: Bias due to incomplete exposure data**	
**Reporting item**	**Your assessment**
Data extracted from study record(s) to support your assessment for this domain	
Rating	*Probably low*
Justification for rating	*Some incomplete information on occupation. But only 26 from the sample had incomplete data and were excluded prior to analyses.*<br><br>*"In practice, 1900 participants were selected to ensure sufficient sample size in the case of non-response or missing information. As a result, 1826 participants were interviewed (i.e. response rate was 96%); however, 26 participants were missed important information (e.g. gender, occupation, utilization of health services, and health insurance). Therefore, 1800 participants were used in data analysis." Page 4*
**Domain 6: Bias due to incomplete outcome data**	
**Reporting item**	**Your assessment**
Rating	*Probably low*
Justification for rating	*Only 26 had missing data on use and were excluded*
**Domain 7: Bias due to selective reporting of exposures**	
**Reporting item**	**Your assessment**
Rating	*No information*
Justification for rating	No information
**Domain 8: Bias due to selective reporting of outcome**	
**Reporting item**	**Your assessment**
Rating	*No information*
Justification for rating	No information
**Domain 9: Bias due to differences between numerator and denominator**	
**Reporting item**	**Your assessment**
Rating	*Low*

Justification for rating	*Outcome (numerator) part of the denominator*<br><br>*Table 1 and table 2 page 13, 15*
**Domain 10: Conflict of interest**	
**Reporting item**	**Your assessment**
Rating	*Probably low*
Justification for rating	*Declaration: Page 1 (page 44 in journal)*

10. Giatti, 2008: Health services use.

**Domain 1: Bias due to selection of participants into the study**	
**Reporting item**	**Your assessment**
Definition of target population	*Working and unemployed men aged 15 to 64 years old in Brazil*
Definition of the study population	*Male participants between 15 and 64 years that participated in the 1998 and 2003 National Household Surveys in metropolitan regions of Brazil.*
Rating	*Probably low*
Justification	*Study sample probably representative of the target population and total male population since it's a National Survey.*<br><br>*"Men aged between 15 and 64 who live in Brazilian metropolitan regions and were included in the economically active population among participants of the 1998 and 2003 National Household Surveys, carried out by Brazilian Institute for Geography and Statistics (IBGE)." Page 2397*
**Domain 2: Bias due to lack of blinding of study personnel**	
**Reporting item**	**Your assessment**
Rating	*Probably low*
Justification for rating	*National Household survey and then stratified by employment status so bias due to blinding may not be a significant issue.*
**Domain 3: Bias due to misclassification of the exposure**	
**Reporting item**	**Your assessment**
Rating	*Probably low*
Justification for rating	*Exposure defined- informal and formal*<br><br>*"The explanatory variable that is of most interest to this study was labor market status, classified in six groups: full-time (≥ 40 hours/ week) employment with social protection; part time (< 40 hours/week) employment with social protection; full time unprotected employment (informed work); part-time unprotected employment (informed work); short term (< 12 months) and long term (≥ 12 months) unemployment. Employment with social protection*

	includes those jobs with a signed working agreement and/or social protection cover. Unprotected employment refers to situations where the employee is not registered or receives no social benefits. "Page 2397
**Domain 4: Bias due to misclassification of the outcome**	
**Reporting item**	**Your assessment**
Rating	*Probably low*
Justification for rating	*Outcome defined but self-reported info and also a respondent may have answered on behalf of another individual but misclassification would have occurred in all groups*<br><br>*"Healthcare use indicators were: medical visit and hospitalization in the past 12 months. Page 2397 &*<br><br>*The information was obtained through interviews, though these were not always given by the individual subject. Page 2397*<br><br>*In the two surveys, information was obtained by household interviews using a proxy informant, when necessary. There seems to be a reasonable agreement between objective health information given by the interviewee himself and by proxy informants". Page 2404*
**Domain 5: Bias due to incomplete exposure data**	
**Reporting item**	**Your assessment**
Rating	*Probably low*
Justification for rating	*No information on missing data but all totals add up. Will affect all groups but unlikely missing data*
**Domain 6: Bias due to incomplete outcome data**	
**Reporting item**	**Your assessment**
Rating	*Probably low*
Justification for rating	*No information- but again no discrepancies in totals. Will affect all groups*
**Domain 7: Bias due to selective reporting of exposures**	
**Reporting item**	**Your assessment**
Rating	*Probably low*
Justification for rating	*Secondary data used from household surveys thus unlikely to selectively not report an exposure- as this exposure was not the primary exposure for consideration in the original survey*
**Domain 8: Bias due to selective reporting of outcome**	
**Reporting item**	**Your assessment**

Rating	*Probably low*
Justification for rating	*Secondary data used from household surveys thus unlikely to selectively not report this outcome as this outcome was not the primary outcome for consideration in the original survey*
**Domain 9: Bias due to differences between numerator and denominator**	
**Reporting item**	**Your assessment**
Rating	*Low*
Justification for rating	*Numerators should be part of the population used in the denominator.*<br><br>*Table 1 page 2398 shows population in each exposure category*
**Domain 10: Conflict of interest**	
**Reporting item**	**Your assessment**
Rating	*Probably low*
Justification for rating	*No evidence of conflict*

11. Giatti, 2011: Health services use.

**Domain 1: Bias due to selection of participants into the study**	
**Reporting item**	**Your assessment**
Definition of target population	*Working and unemployed men aged 15 to 64 years old in Brazil*
Definition of the study population	*Male participants between 15 and 64 years that participated in the 1998, 2003 and 2008 National Household Surveys in metropolitan regions of Brazil.*
Rating	*Probably low*
Justification	*Study population probably representative of the male population as used data from a National survey*<br><br>*"The present study intends to compare the of health services in men economically according to the market situation in the years 1998, 2003 and 2008, and to investigate if both are associated after adjustment by socio-demographic characteristics and by a indicator of health need. The population study was restricted to men because there are recognized gender differential in illness, perception of health and use of services and insertion in the labor market. Men, in particular those of working age, despite the greater risk of illness and death, use less health services than women. "*
**Domain 2: Bias due to lack of blinding of study personnel**	
**Reporting item**	**Your assessment**
Rating	*Probably low*

Justification for rating	*National Household survey and then stratified by employment status so bias due to blinding may not be a significant issue.*
**Domain 3: Bias due to misclassification of the exposure**	
**Reporting item**	**Your assessment**
Rating	*Probably low*
Justification for rating	*Exposure defined- informal and formal*<br><br>*"The explanatory variable of interest was the in the labor market, categorized as work with social protection, unprotected work social and unemployment. Work with protection social responsibility corresponds to working with signed or only with contribution to social security Social. Work without social protection is the one without a signed and no contribution for social security. Unemployment corresponds to the condition of individuals who did not work and searched for work in the week of reference adopted by PNAD." (from the translated document page 8)*
**Domain 4: Bias due to misclassification of the outcome**	
**Reporting item**	**Your assessment**
Rating	*Probably low*
Justification for rating	*Outcome defined but self-reported info and also a respondent may have answered on behalf of another individual but misclassification would have occurred in all groups*<br><br>*To describe some usage characteristics health services were added to the variables look for the same health service (yes, no) obtained by the question Usually looking same place, same doctor or even service when you need Cheers? and type of service you are looking for (health, private practice, outpatient clinics include outpatient clinic or office or union, outpatient clinic or clinic and outpatient clinic or hospital office, emergencies and others that brings together pharmacy, community service and other type of service) When you are sick or need health services they seek ....of these, we also include coverage per plan (yes, no) and the payer of the health plan. (Page 9 of translated doc)*
**Domain 5: Bias due to incomplete exposure data**	
**Reporting item**	**Your assessment**
Rating	*Probably low*
Justification for rating	*No information on missing data, but no missing data reflected in numbers. Will affect all groups*
**Domain 6: Bias due to incomplete outcome data**	
**Reporting item**	**Your assessment**
Rating	*Probably low*
Justification for rating	No information on missing data. Totals add up. Will affect all groups
**Domain 7: Bias due to selective reporting of exposures**	
**Reporting item**	**Your assessment**

Rating	*Probably low*
Justification for rating	*Secondary data used from household surveys thus unlikely to selectively not report an exposure as this exposure was not the primary exposure for consideration in the original survey*
**Domain 8: Bias due to selective reporting of outcome**	
**Reporting item**	**Your assessment**
Rating	*Probably low*
Justification for rating	*Secondary data used from household surveys thus unlikely to selectively not report an exposure- as this exposure was not the primary exposure for consideration in the original survey*
**Domain 9: Bias due to differences between numerator and denominator**	
**Reporting item**	**Your assessment**
Rating	*low*
Justification for rating	*Numerator should be part of the denominator. See table 1 and 2*
**Domain 10: Conflict of interest**	
**Reporting item**	**Your assessment**
Rating	*Probably low*
Justification for rating	No evidence of conflict

12. Miquillan, 2013: Health services use.

**Domain 1: Bias due to selection of participants into the study**	
**Reporting item**	**Your assessment**
Definition of target population	*Male and female workers from 18-64 years old- National Survey of sample of households (PNAD/2008)*
Definition of the study population	*Male and female workers from 18-64 years old- National Survey of sample of households (PNAD/2008)*
Rating	*Low*
Justification	*Assessing occupational injuries in formal vs informal workers was not the primary purpose thus it is unlikely that selection bias would have occurred and if present will affect all groups. Similar to census data.*<br><br>*"A cross-sectional study with secondary data basic research and supplementation of the PNAD / 2008. The PNAD is by the Brazilian Institute of Geography and Statistics (IBGE), being representative of the population residing in the urban domiciliary and rural areas of the capitals, metropolitan Federal (n = 391,868 people and 150,591 units domiciliary). It is a probabilistic sample of households in three stages of selection: primary units (counties);*

	secondary units (sectors censuses); and tertiary units / units domiciles (private households and housing in collective households). Excluded street dwellers, people living in embassies, consulates, legacies and residents in institutional establishments."
**Domain 2: Bias due to lack of blinding of study personnel**	
**Reporting item**	**Your assessment**
Rating	*Probably low*
Justification for rating	*The exposure and outcome of this study was not the primary objective, thus blinding of staff unlikely to influence result*
**Domain 3: Bias due to misclassification of the exposure**	
**Reporting item**	**Your assessment**
Rating	*Probably low*
Justification for rating	*Exposure is defined.*<br><br>*"Among the economically active people, selected men and women between 18 and 64 employed in paid employment agricultural or unoccupied in the reference week of the research (n = 152,322). Page 6*<br>*According to the information available in the PNAD, the formal work is that exercised through contract (governed by Consolidation of Labor Laws) or statutory; informal workers are those without employment relationships, regardless of contribution paid by the worker. The unemployed are those who did not any activity, but sought occupation in the*<br>*reference period." Page 6/7*
**Domain 4: Bias due to misclassification of the outcome**	
**Reporting item**	**Your assessment**
Rating	*Probably low*
Justification for rating	*Specific questions related to access and use*<br><br>*"The characteristics of health, access and use of health services were considered variables and can be modified by virtue of the situation of the worker in the labor market and their socio-demographic characteristics and and economic development. The main determinant variable of the use of health services is the demand for care, being the health conditions (referred health, withdrawal and bed rest) determinants of the need for demand … Also had specific questions related to access and use". page 7/8*
**Domain 5: Bias due to incomplete exposure data**	
**Reporting item**	**Your assessment**
Rating	*No information*
Justification for rating	*No information*
**Domain 6: Bias due to incomplete outcome data**	

Reporting item	Your assessment
Rating	*No information*
Justification for rating	No information
**Domain 7: Bias due to selective reporting of exposures**	
**Reporting item**	**Your assessment**
Rating	*Probably low*
Justification for rating	*If there was poor reporting – it will affect all groups*
**Domain 8: Bias due to selective reporting of outcome**	
**Reporting item**	**Your assessment**
Rating	*Probably low*
Justification for rating	*If there was poor reporting – it will affect all groups since this is based on secondary data and fieldworkers were not aware of the outcome at data collection*
**Domain 9: Bias due to differences between numerator and denominator**	
**Reporting item**	**Your assessment**
Rating	*low*
Justification for rating	*Tables show the numerator and denominator Table 1 & 2*
**Domain 10: Conflict of interest**	
**Reporting item**	**Your assessment**
Rating	*Probably low*
Justification for rating	*No evidence*

Table S3. Quality of Evidence.

No.	Certainty Assessment							No. of participants	Effect	Quality of evidence [#]	Importance [##]
	No of studies	Study Design	Risk of bias [a]	Inconsistency [b]	Indirectness [c]	Imprecision [d]	Other Considerations [e]		RR/OR/PR (95% CI)		
1.	**Health service utilisation**										
	4	Cross-sectional studies<br><br>= low quality	Low –all four were rated low or very low probability for all domains. One study had a probably high for selection bias and misclassification of exposure. But no serious limitations thus No down grade	The I² =89% Indicating substantial heterogeneity.<br><br>Downgrade by 1	1.Different populations- 3 in Brazil and 1 in Vietnam. No populations accessed in high income countries. 2. Gender disparities- 2 studies had male only participants<br><br>Downgrade by 1	The overall 95% CI was not wide<br><br>No downgrade	Not applicable- Publication bias not conducted due to the low number of studies for the outcome.	195667	OR 0.89 (0.85-0.94)<br><br>Low OR Downgrade by 1	⊕⊕◯◯	Critical<br><br>Lack of access to health facility can be life threatening.
2.	**Fatal occupational injuries**										
	2	Cross sectional studies= low quality	High- both studies had high probability of bias in at least one domain<br><br>Downgrade by 2	The I²=95% Downgrade by 1	Only South American country studies available, thus geographically limited.<br>Downgrade by 1	The overall 95% CI is narrow BUT crosses 1<br><br>Downgrade by 1	N/A	More than 1318563 Mora et al., total study participants unknown.	OR 0.65 (0.32-1.32)<br><br>Low OR Downgrade by 1	⊕◯◯◯	Critical due to outcome
3	**Non- fatal occupational injuries**										
	3	Cross sectional studies= low quality	High risk: Multiple domains with probably high risk of bias.<br>Downgrade by 1	I²= 94% Downgrade by 1	Different occupational groups in the different studies.<br>Geographically limited.<br>Downgrade by 1	Wider 95% CI<br><br>Downgrade by 1	N/A	3465	OR 1.57<br>Low OR<br><br>Downgrade 1	⊕◯◯◯	Important but not critical
4	**Depression**										
	3	Cross-sectional studies =low quality	Low risk: majority of the domains very low to low probability<br>No downgrade	I²=87%<br><br>No downgrade	Limited countries involved. Very specific occupations with limited generalisability.<br>Downgrade by 1	Wide 95% CI	N/A	26260	OR 5.02<br><br>HIGH OR = no upgrade since downgraded in two other domains	⊕⊕◯◯	Important but not critical
5	**Musculoskeletal Pain**										

1	Cross sectional studies= low quality	Low risk<br><br>No downgrade	N/A only one study thus no meta-analyses conducted	Geographically limited with a very specific occupational group<br>Down grade by 2	Narrow 95% CI but crosses 1<br><br>Downgrade by 1	N/A	881	1.1<br>Down grade by 1	⊕○○○	Important but not critical

[a] Risk of Bias: To assess risk of bias, we used a modified version of the RoB-SPEO tool for assessing risk of bias in prevalence studies in occupational health (*24*). [b] Inconsistency: heterogeneity was considerable with $I^2 >$ 75% for all studies in which it was measured, and hence all outcomes which measured I^2 were downgraded on this factor. Rule of thumb: https://gdt.gradepro.org/app/handbook/handbook.html#h.j8tx801skdu3 < 40% may be low 30-60% may be moderate 50-90% may be substantial 75-100% may be considerable. [c] Indirectness: all outcomes were restricted geographically (few countries) which resulted in downgrades of quality of evidence for this factor. [d] Impression: excepting Health service utilisation, quality of evidence was downgraded for outcomes because the 95% CI spanned 1. [e] Factors that could increase the quality of evidence were not considered because downgrades were done on other factors.

# Definitions for ratings of the certainty of the evidence.

Ratings	Definitions
⊕⊕⊕⊕<br>High	This research provides a very good indication of the likely effect. The likelihood that the effect will be ***substantially different is low.***
⊕⊕⊕○<br>Moderate	This research provides a good indication of the likely effect. The likelihood that the effect will be substantially different is moderate.
⊕⊕○○<br>Low	This research provides some indication of the likely effect. However, the likelihood that it will be substantially different (a large enough difference that it might have an effect on a decision) is high.
⊕○○○<br>Very Low	This research does not provide a reliable indication of the likely effect. The likelihood that the effect will be substantially different (a large enough difference that it might have an effect on a decision) is very high.

##Importance rating:.

Rating Scale:								
1	2	3	4	5	6	7	8	9
of **least** importance								of **most** importance
of limited importance for making a decision (not included in evidence profile)			important, but not critical for making a decision (included in evidence profile)			Critical for making a decision (included in evidence profile)		

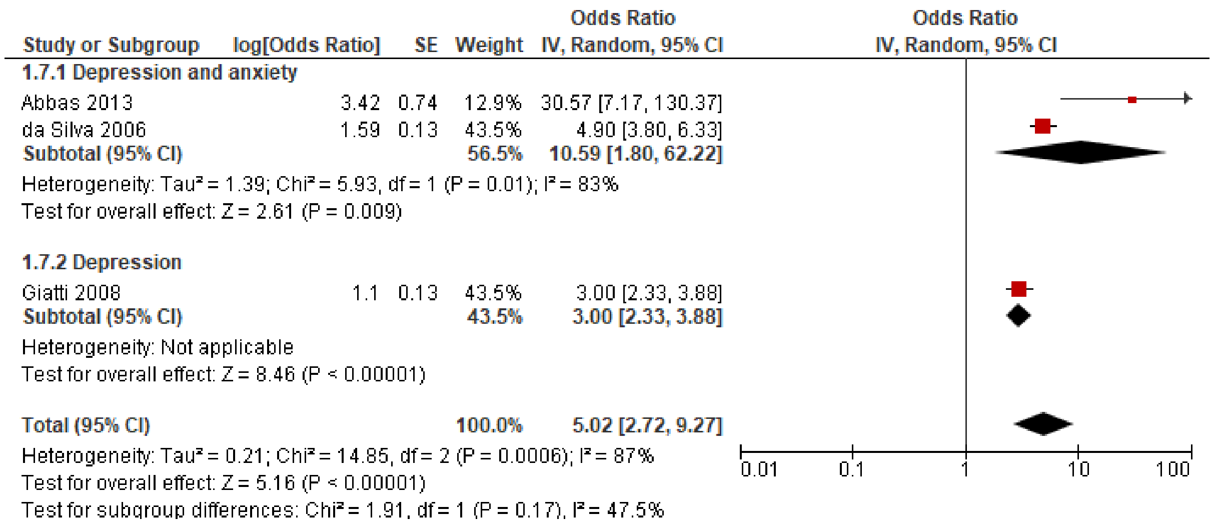

Represents the size of the study

Represents the point estimate and confidence intervals of all the individual studies combined

Figure S1. Sub group analyses for Depression and Anxiety, and Depression alone.
